# Yerba Mate* (Ilex paraguariensis)* Beverage: Nutraceutical Ingredient or Conveyor for the Intake of Medicinal Plants? Evidence from Paraguayan Folk Medicine

**DOI:** 10.1155/2018/6849317

**Published:** 2018-03-14

**Authors:** Monika Kujawska

**Affiliations:** Institute of Ethnology and Cultural Anthropology, University of Lodz, ul. Lindleya 3/5, 90-131 Lodz, Poland

## Abstract

The use of medicinal plants mixed with yerba mate* (Ilex paraguariensis)* has been poorly studied in the ethnopharmacological literature so far. The Paraguayan Mestizo people have the longest tradition of using the yerba mate beverage, apart from the indigenous Guarani people. This study analyses the role of yerba mate and medicinal plants in the treatment of illnesses within Paraguayan folk medicine. The research was conducted among 100 Paraguayan migrants living in Misiones, Argentina, in 2014 and 2015. Yerba mate is not considered to be a medicinal plant by its own virtues but is culturally a very important type of medicinal plant intake. Ninety-seven species are employed in hot and cold versions of the yerba mate beverage. The most important species are as follows:* Allophylus edulis* (highest number of citations),* Aristolochia triangularis* (highest relative importance value), and* Achyrocline flaccida* and* Achyrocline tomentosa *(highest score by Index of Agreement on Species). The plants are used in the treatment of 18 medicinal categories, which include illnesses traditionally treated with plants: digestive system, humoral medicine, and relatively new health conditions such as diabetes, hypertension, and high levels of cholesterol. Newly incorporated medicinal plants, such as* Moringa oleifera, *are ingested predominantly or exclusively with the* mate* beverage.

## 1. Introduction

Yerba mate (*Ilex paraguariensis *A.St.-Hil., Aquifoliaceae) is a native tree growing in the subtropics of South America, present in Southern Brazil, Northeastern Argentina, Eastern Paraguay, and Uruguay [1]. The yerba mate beverage has been consumed traditionally by Guarani indigenous people since before the conquest of South America by the Spaniards [2]. The commercial potential of this plant was discovered by the Jesuits, who brought wild growing yerba mate into cultivation. Pedro de Montenegro, a Jesuit monk, in his* Materia Medica Misionera* described the use of the most important species for the Guarani people, in which yerba mate appeared on the top of the list [3]. The Guarani name for yerba mate is* ka'a* which means “a plant” or “a herb”; hence yerba mate has been considered by this group as the plant* par excellence* [3]. Yerba mate was also known as Jesuit tea or Paraguayan tea and shipped as such to Europe [2]. With the expulsion of the Jesuits in 1768, the plantations went wild. By this time, the yerba mate beverage was already popular among Mestizo people (of Spanish and Guarani origin). Since the end of the 19th century, it also became a daily beverage for the European migrants who partly colonized Southern Brazil, Northeastern Argentina, and, to a lesser extent, Eastern Paraguay [4]. Nowadays yerba mate is consumed at the rate of more than one litre per day by millions of people in the above-mentioned countries [4, 5]. It plays a very special social role and constitutes a very important form of caffeine intake [2, 4, 5]. Its popularity is also increasing outside South America due to its pharmacological properties, proven to be beneficial to health [4, 6, 7]. It is also a very important drink in Syria and Lebanon due to Syro-Lebanese migration to Argentina in the second half of the 19th century. Many migrants who returned to the Levant in the 1920s took the habit of drinking* mate* with them [8, 9].

Over the last 20 years there has been an increase in studies of the pharmacologic properties of* Ilex paraguariensis*, which have been reviewed [4, 6, 7, 10]. Numerous active compounds have been identified in yerba mate. Phenolic compounds predominate caffeoyl derivatives (caffeic acid, chlorogenic acid) [11, 12], xanthines (caffeine and theobromine), which are a class of purine alkaloids found in many other plants such as tea and coffee, flavonoids (quercetin, kaempferol, and rutin), and tannins [7]. Numerous triterpenoid saponins have also been identified, including those derived from ursolic acids known as metasaponins [4, 7]. Saponins are responsible for the distinct flavour of yerba mate extracts [7]. Yerba mate also contains minerals (P, Fe, and Ca) and vitamins (C, B1, and B2) [13].

Research on extracts and isolated compounds from yerba mate has provided a number of pharmacological applications. Studies have demonstrated that yerba mate leaves have antioxidant [11], antiobesity [14, 15], antidiabetic, digestive improvement and cardiovascular properties [16, 17], and chemopreventative ones (preventing cellular damage that may cause chronic diseases) [18]. The consumption of yerba mate infusion reduces LDL-cholesterol in parallel with an increase in HDL-cholesterol, as observed in studies on humans [19]. Yerba mate extract also reduces acute lung inflammation, as observed in the animal model [4]. Antimicrobial activity of* Ilex paraguariensis* has been recently studied as well [20].

Some ethnobotanical studies from the south cone of South America report medicinal uses of yerba mate beverage [21, 22]. Few ethnobotanical and ethnopharmacological studies mention that various medicinal plants are consumed together with the yerba mate beverage by Mestizo and European migrants living in Argentina and Paraguay [23–26]. However, very little is known about how medicinal plants are combined with yerba mate beverage by local people. Additionally, medicinal plant use by Paraguayan Mestizo people is poorly documented in the English-language scientific literature, with very few exceptions [23, 26–30]. The documentation of medicinal plants and analysis of traditional knowledge related to the yerba mate beverage by Paraguayan Mestizo people is of paramount importance for two reasons: (1) apart from indigenous Guarani peoples, they have the longest tradition of using yerba mate and mixing it with medicinal plants; (2) The Paraguayan people are described in the literature as knowledgeable about medicinal plants [30, 31]. Nearly 80% of the population of Paraguay consume medicinal plants on a daily basis [30]. However, the relationship between traditional uses and pharmaceutical properties is poorly studied.

The objectives of this contribution were to (1) document and analyse the role of yerba mate in prophylaxis and treatment by Paraguayan Mestizo people; (2) evaluate the role of medicinal plants in yerba mate beverages, and (3) describe the scope of illnesses treated with yerba mate beverage and medicinal plants. Additionally, two questions guided my research and analysis: (1) Does any pattern exist showing that particular illnesses are treated with a hot version of yerba mate beverage and others with a cold one? (2) How receptive is this traditional mode of plant administration to new health challenges and new medicinal plants, previously unknown to the Paraguayan people?

## 2. Material and Methods

### 2.1. Study Area and Ethnographic Setting

The research was conducted among Paraguayan migrants living in the province of Misiones, Argentina. Misiones borders with Paraguay and Brazil and is primarily a semideciduous Atlantic Forest. The Atlantic Forest is one of the most diverse ecosystems in the world [32]. The climate is subtropical humid with no dry seasons, a mean annual rainfall of 2,000 mm, and a mean annual temperature of 20°C [33]. Currently, nearly 40% of original forest cover is preserved in Argentina, in contrast to Brazil and Paraguay, where it comprises approximately 8% [34]. The flora of Misiones comprise 3000 vascular plants, of which more than 500 species have been used for phytotherapeutic purposes by the different ethnic groups living there: the indigenous Guarani people, Mestizos of different origins (Argentinean, Paraguayan, and Brazilian) and European migrants [35].

The colonial policy in Paraguay, which was based not on mineral extraction but on settlement, resulted in the early emergence of an entirely new Paraguayan Mestizo culture, neither Spanish nor Guarani, but influenced by both of them [36, 37]. Within Mestizo culture, the Guarani language was preserved, as well as native customs and knowledge. Paraguayan Mestizo people have had the contact with European phytotherapeutic traditions since early colonial times, when they were introduced by Catholic nuns and monks, who promoted the use of well-known medicinal plants from the Mediterranean region, such as* Artemisia*,* Ruta*,* Mentha,* and* Rosmarinus*, as well as Asian species, such as* Cymbopogon citratus* and* Citrus* spp. [38]. The basic Hippocratic and humoral concepts were introduced through the same channels [39, 40].

Since the 19th century,* Criollos* (the local name for Mestizos) have been coming to Misiones from Paraguay. According to the 2010 census, the Paraguayan community in Argentina accounts for 550.713 persons. This figure represents 30.5% of all immigrants in the country and approximately 8.6% of the total population of Paraguay [41]. The push and pull factors for migration were both economic and political in character. Paraguayan migrants have been regarded as having low formal educational levels, especially those migrating to Argentina in the 1970s, who comprise the majority of the study population. In Misiones, Paraguayan people found employment in the agricultural and forestry sectors.

Given that in Misiones the natural environment is generally better preserved than it is in Paraguay [32], Paraguayan migrants originating from the same ecoregion of the Atlantic Forest have presumably found better conditions for implementing and transmitting their traditional ethnomedicine than they did in their own country of origin.

### 2.2. Data Collection

The research was conducted among 100 Paraguayan migrants, 61 women (aged between 30 and 95, mean 61) and 39 men (aged between 28 and 90, mean 61,5). Thirty-two participants were born in Misiones, Argentina, while 68 hailed from the Eastern provinces of Paraguay within the same ecoregion of the Atlantic Forest. The data were collected during two phases of fieldwork, lasting three months each in 2014 and 2015. The free listing technique was applied [42]. Each informant was asked to name all the medicinal plants she/he knew and used (emphasis was placed on personal experience). When a list of names was produced, further questions were asked relating to parts used, medicinal uses, forms of administration, and modes of obtaining the plants.

**Figure 1:**
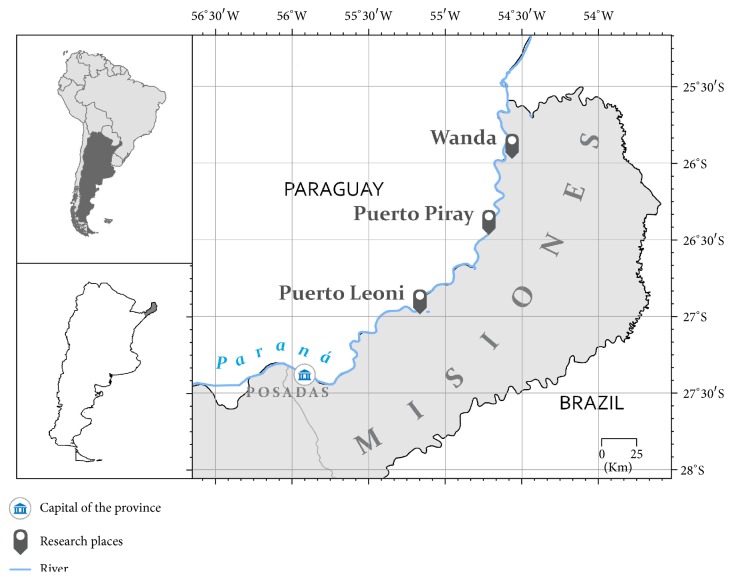
Distribution of the study localities, Misiones, Argentina.

The research took place in three localities, on a gradient from rural to urban, with the semirural component predominating. The three localities—Wanda with Puerto Wanda as one unit, Piray Km 18 (Puerto Piray municipality), and Puerto Leoni—are situated on the bank of the Parana River, the border between Argentina and Paraguay (Figure 1). In general terms, the study participants can be described as small scale farmers working on 1-2-hectare plots* (chacras)*, where they cultivate staple crops such as manioc and maize. In addition, male participants usually find off-farm employment, mainly in the forestry industry.

The documented botanical taxa are supported with voucher herbarium specimens stored in the CTES herbarium in the Instituto de Botánica del Nordeste (IBONE), Corrientes, Argentina, and in the herbarium of the Museo Argentino de Ciencias Naturales “Bernardino Rivadavia” in Buenos Aires (BA). Prior to each interview, study participants gave oral informed consent for their participation. There was no requirement for the project to pass any ethical commission, either in Argentinian or in Polish institutions, as the informants were not subject to any other treatment than voluntary interviews.

### 2.3. Data Analysis

In order to analyse the scope of medicinal categories, which include body systems, and determine the most important illnesses treated with yerba mate extracts, two measures were used as a proxy: number of species and use reports (UR). A use report is defined here as a description by an informant (i) of species (s) used for a medicinal purpose (p); hence two different medicinal uses of a given species reported by the same informants are counted as two use reports [43].

The relative importance (RI) value was calculated for each plant species based on the normalized number of medicinal uses attributed to it and the number of body systems/medicinal categories it treated [38]. Relative importance is a versatile index, which shows the extent of medicinal application of a given species. The reasons for versatile use of a species may be very diverse; for example, this may reflect a trial-and-error effort—a constant search for accurate or new medicinal applications for some widely available species. Taking this into account, another index was also used, the Index of Agreement on Species (IAS) [43], to measure the consistency of medicinal uses of a given species. This is a modified version of Index on Agreement of Remedies developed by Trotter and Logan [44].

The Sørensen similarity coefficient was used to check whether similar plants were used in the treatment of apparently similar illnesses. The formula is as follows: Ss = 2*a*/(2*a* + *b* + *c*), where *a* is number of shared species, *b* is number of exclusive species in group 1, and *c* is number of exclusive species in group 2. The result is then multiplied by 100, in order to express it as a percentage.

## 3. Results and Discussion

### 3.1. Social and Medicinal Context of Drinking* Mate* and* Tereré*

The sources describe four different forms of drinking processed minced leaves and stems of yerba mate [10], but the representatives of the Paraguayan community in Misiones use two basic forms of ingesting it—a hot version called* mate* and a cold one:* tereré*. In general terms,* mate* is drunk all the year round, usually in the morning and in the afternoon. On hot summer days the afternoon* mate* may be replaced by* tereré*. Additionally,* tereré* may be drunk at noon, before lunch, and during the summer (December–March). On a few occasions, male informants mentioned that they would drink* tereré* the whole year round, independently of the weather. These observations stay in line with reports from rural areas of Paraguay [23, 26].

The hot beverage,* mate*, is normally prepared in a gourd (*Lagenaria* spp.) also called* mate*. A metal straw,* bombilla*, is used to ingest the extract. Industrialized yerba mate is always chosen, preferably the one with stems* (con palo)*. According to the informants, such yerba mate does not wash away rapidly and less powder is ingested, compared to yerba mate without stems* (sin palo)*. Small amounts of hot water (77–80°C) are repeatedly poured over a serving, ca. 50 g of packed processed yerba mate. Yerba mate may be poured from a thermos or directly from a steel kettle. The second method normally takes place in the kitchen with a stove, where the kettle can be heated up.

**Figure 2:**
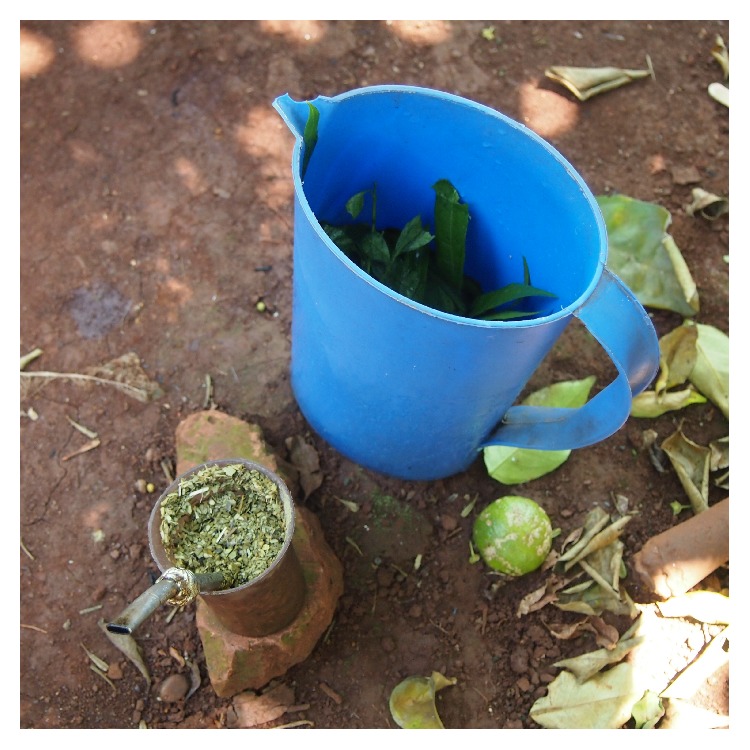
*Tereré* with* kokû* leaves* (Allophylus edulis)* prepared for a noon round on a hot summer day (Piray Km 18, Puerto Piray Municipality, Misiones, 2014).

The cold beverage—*tereré*—is drunk virtually from any container. Normally plastic, metal, or glass cups are used or those made from bamboo-like plants (*Chusquea* spp.). A metal or bamboo* bombilla* is used. Another bigger container is filled with cold water and some other additives (Figure 2). In this case, industrially processed yerba mate is also used, but there is also* yerba canchada—*especially processed for drinking* tereré*, in which leaves are crushed into large pieces.

**Figure 3:**
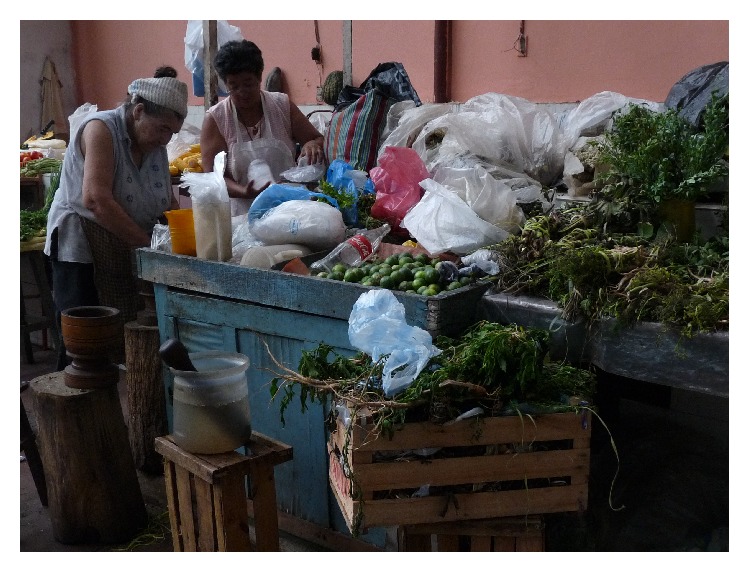
Medicinal plants, additives to* tereré*, and mortars in the Asunción market.

The medicinal plants used with* mate* are placed in a thermos or in the gourd. Whenever hard plant parts are used, such as bark or roots, they are placed in a thermos with hot water or in a kettle. Leaves, stems, and flowers are placed directly in the gourd with yerba mate. Some Paraguayans also add sugar to* mate—*a spoonful is added to the yerba mate every round or every second round (the* mate* server starts from her/himself and then pours water into a gourd for each person in a clockwise order). When the* tereré* is prepared, all the medicinal plants are placed in a container with cold water, and sometimes ice is added. If time permits, plants are left for several minutes to macerate. Sometimes the maceration process is accelerated, by squeezing leaves in the hands to stimulate the release of juices into the water. I have never, however, observed the use of mortars to crush medicinal plants before adding them to cold water in order to prepare the medicinal maceration for* tereré—*something widely practiced in Paraguayan cities, for example, Asunción and Encarnación (Figure 3).

Drinking yerba mate is social behaviour* par excellence*. People avoid drinking* mate* or* tereré* on their own. The afternoon* mate*, especially, is shared with family, neighbours, friends, and other visitors. Sometimes the addition of medicinal plants is negotiated among the participants, and in other situations the invited persons ingest the plants which the host is currently drinking with his/her yerba mate. Of anecdotal character is the story told by local doctors that Mestizo women (not necessarily of Paraguayan origin) in Misiones would add contraceptive pills to* mate* and then drink it with family and relatives.

Yerba mate itself is not perceived as a medicinal plant by the study community. Just one person mentioned its sole use as medicinal. Nor is it conceived as a nutraceutical ingredient; at least this was never expressed explicitly by informants. Hence, it is the medicinal plants, used together with* mate* or* tereré,* that determine the medicinal properties of a yerba mate beverage. Occasionally, plants are used to improve the taste or give a flavour to yerba mate without clear medicinal cues; rosemary* (Rosmarinus officinalis)*, mint (*Mentha* spp.), and lemon grass* (Cymbopogon citratus)* are used in this way. The importance of the yerba mate beverage as a vehicle for medicinal plants intake was also reported in another study from Paraguay. Goyke [26] stated that, for all the top scoring plants, yerba mate was either the typical or the exclusive way people consumed them. This shows how important yerba mate is medicinally, though not by its own virtue. In this sense, these findings differ from what is reported in pharmacological literature [7].

Some epidemiological studies have reported an association between the consumption of yerba mate and increased risk of various types of cancer, including oral, laryngeal, and bladder cancer [7, 45, 46]. During my field campaign in 2014 this message was spread by radio, television, and newspapers to the general audience in Argentina. Information about the potential carcinogenic effects of drinking yerba mate produced serious concern among informants about the future of their habit. Nonetheless, the subsequent message that spread rapidly among them was that drinking* mate* too hot might produce cancer, which was heard with deep relief.

### 3.2. Characteristics of Plants Used with* Mate* and* Tereré*

The Paraguayan community in Misiones uses 97 medicinal plant species (552 UR) together with* mate* (58 species),* tereré* (17), or both (22) (Table 1). Of these 97 species, 63 are native, 30 introduced, and four naturalized. Most of the plants come from cultivation (43), followed by species gathered from the wild (39); however 12 species have been reported as cultivated by some informants and collected from ruderal areas by others. Only four species were purchased in the market or from Paraguayan ambulatory sellers—women who bring plant for sale from Paraguay through the border on the Parana River.

**Figure 4:**
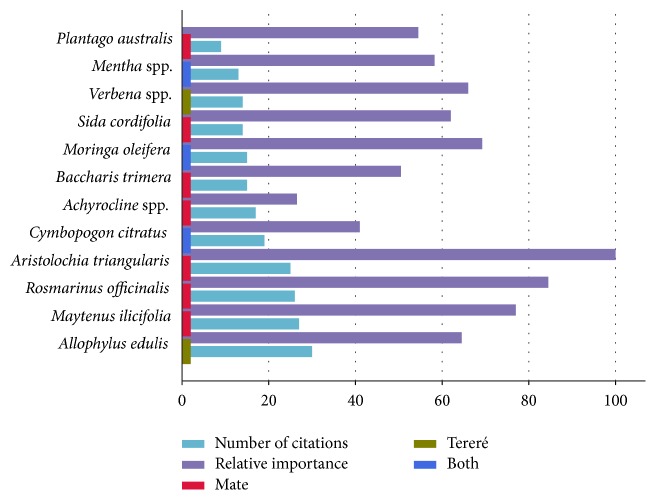
Species with the highest number of citations and relative importance value.

*Allophylus edulis*,* kokû*, is the plant with the highest number of citations (30) and UR (34) (Figure 4). It is used only with* tereré* and is a “cold” remedy* par excellence* for Paraguayan people. It is also widely used in the treatment of digestive tract illnesses, hepatitis, and hypertension. Some pharmacological properties of this species are known, which largely coincide with the folk use, according to which* A*.* edulis* acts as a hepatoprotector (flavonoids) and angiotensin converting enzyme (ACE) inhibitor [47].

The most versatile plant species (with the highest RI value) is* Aristolochia triangularis*,* ysypo milhombre(s)*. This species is only applied in* mate*. It is used in the treatment of nine different categories, the most important being humoral medicine (blood cleansing), but also urinary (infections) cardiovascular (hypertension), reproductive, digestive, and musculoskeletal (rheumatism) systems, and for general infections. Its use in the reproductive health category is unclear. For some informants,* A. triangularis* is a contraceptive or an abortifacient agent, when used in greater doses, and for others it cures venereal diseases and enhances male potency—this latter information is inscribed in the plant name* ysypo* [liana]* milhombre(s)* [thousand-men]. All informants agreed that it should be used with caution; however they could not explain what the exact side effects might be. Given its popularity among the Paraguayan community, there is surprisingly little information published on the folk medicinal use of* A*.* triangularis*, just two reports from Argentina [48, 49], one from Brazil [50], and a few general ones from South America [51]. The only pharmacologically tested properties found so far are cytotoxic [49]. The known chemical compounds are alkaloids, essential oils, lignans, terpenoids, saponins, tannins, and aristolochic acids (AAs) [52–54]. In the ethnopharmacological review by Heinrich et al. [51], different* Aristolochia* spp. were described, including* A*.* triangularis*. Since it also contains aristolochic acids, the consumption of the species poses a risk. In China and Europe species of* Aristolochia *have been associated with nephropathy, but there is a lack of data about the potential risk of renal toxicity from consuming AAs in South American literature [51].

The species with the highest consistency and consensus on use, measured by Index of Agreement on Species (IAS), were those with just one use mentioned by two or three respondents. One of the taxa with a parallel high number of citations and IAS score was* Achyrocline* spp. There are two species used indiscriminately within this genus:* Achyrocline flaccida *and* Achyrocline tomentosa *called* jate'i ka'a* and* marcela*. These taxa are used for digestion problems and as “hot” remedy and they are always applied in* mate* by Paraguayan people. The importance of* Achyrocline* species in Paraguayan folk medicine was already highlighted by Schmeda-Hirschmann and Bordas [23]. According to pharmaceutical studies,* A*.* flaccida *possesses antiviral and antibacterial activity [55, 56]. It contains caffeoyl derivatives, essential oils, flavonoids, and sesquiterpenes [57]. Phytochemical studies of* A*.* tomentosa* reported the presence of flavonoids as well as 27 mayor and trace elements [58, 59].

Other species from the list of the most cited ones and with the highest RI value, include native plants such as* Maytenus ilicifolia*,* Baccharis trimera*,* Sida cordifolia,* and* Verbena litoralis*/*Verbena montevidensis—*used indiscriminately. All of them have been studied phytochemically [60]. There are also four introduced plants on this list, all of them well studied:* Rosmarinus officinalis*,* Cymbopogon citratus,* and* Mentha* spp. with a long tradition of use and* Moringa oleifera* which has only recently (in the last 5-6 years) been incorporated by Paraguayan migrants in Misiones.

How many antidiabetic properties can be found in the plants used to treat diabetes by Paraguayan people? The study community uses 17 different plant species with* mate* and* tereré *to treat diabetes mellitus type 2. From this group only* Moringa oleifera* [61, 62] and* Scoparia dulcis* [63] have been confirmed to possess antidiabetic properties. Interestingly, Paraguayan migrants, through their family and friendship channels, obtain seedlings of* Cissus verticillata* and* Smallanthus connatus* from Paraguay, as potent antidiabetic plants.* C*.* verticillata* is called* insulina* by them; however so far only antifungal activity has been ascribed to this species [64]. Therefore, only a small portion of the plants used as antidiabetic by Paraguayan people have been proven to possess pharmacologically tested properties for diabetes. Most probably the pharmacological action in the treatment of this health problem may be ascribed directly to* Ilex paraguariensis,* while informants see it in the properties of medicinal plants used with yerba mate. The question here may be whether medicinal plants with pharmacologically tested antidiabetic properties have an additive or synergetic function in the beverage.

A similar question was posed in the context of hypertension—another chronic problem for which the Paraguayan community in Misiones uses 19 different species. From this list, only* Eugenia uniflora* has been assigned clear hypotensive activity [65, 66]. Indeed, this species is applied in* mate* and* tereré* with the highest frequency, with the function of decreasing blood pressure.

These two health problems have been incorporated into phytotherapy most recently by the Paraguayan people, and comparison with the pharmaceutical literature shows that Paraguayan people are still in the process of using trial-and-error to find adequate herbal medicines, efficacious in the treatment of these health conditions. The relatively high number of species used for these events also indicates a personalistic approach to the search for the right drug.

### 3.3. Characteristics of Illnesses Treated with Mixtures Based on the Yerba Mate Beverage

**Figure 5:**
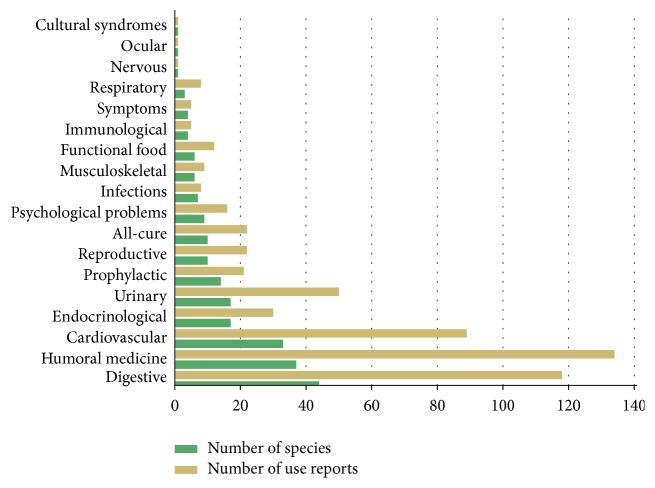
Medicinal categories, including body systems, for which medicinal plants are used together with the yerba mate beverage.

Medicinal plants are used for their preventive and curative properties. The scope of illnesses treated with medicinal plants used in* mate* and* tereré* is very wide. Eighteen use categories have been reported, including 10 body systems (Figure 5). The most important categories are digestive, followed by humoral medicine, cardiovascular, endocrinological, and urinary categories. Interestingly, respiratory system disorders are rarely treated in this way (eight use reports for respiratory tract illnesses). The lack of dermatological problems is understandable—these ailments are treated mainly by the external application of medicinal plants.

**Figure 6:**
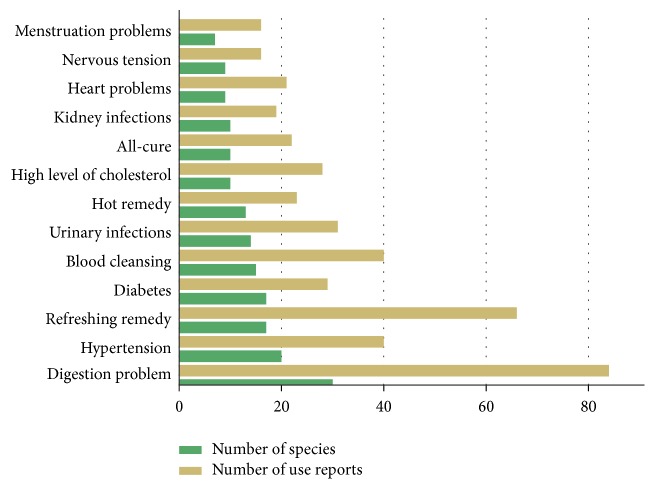
Particular illnesses treated with the highest number of species.

The illnesses treated with the highest number of species are digestive problems (Figure 6). On one hand, emerging illnesses and symptoms such as hypertension, diabetes, and high levels of cholesterol are among the most important health problems treated with plants and yerba mate beverage. On the other hand, old humoral imbalances such as “hot” and “cold” syndromes and blood cleansing—another Hippocratic concept—are treated in this way. Other illnesses treated with a relatively high number of plants are urinary and kidney infections, heart problems, and nervous tension and finally menstrual problems related to women's reproductive health.

Blood cleansing is an old concept, widely used by the Paraguayan people. According to informants, it is desirable to clean the blood periodically from impurity and to make it more fluid—thick blood is considered a symptom of illness. Medicines used for high levels of cholesterol and diabetes mellitus type 2 act on a similar conceptual basis as blood cleansers for the Paraguayan people. Therefore, my working hypothesis was that, due to this conceptual similarity, Paraguayan people would adopt plants used for blood cleansing to treat new illnesses: diabetes and high levels of cholesterol. This assumption turned out to be wrong, as informants clearly distinguished the differences between these three health problems (though all concentrated in the blood). Actually, the similarities of plants used for blood cleansing and diabetes are at 19% and for blood cleansing and high levels of cholesterol are at 32% and between diabetes and high levels of cholesterol are at 52%, meaning that if any cluster of plants exists, it only occurs between diabetes and high levels of cholesterol. Diabetes, of these three health problems, is treated with the greatest number of plants. Seventeen different species (29 UR) have been reported, but most of them were cited by single respondents, with only* Smallanthus conatus* and* Sorocea bonplandii* mentioned by 4 participants. Blood cleansing is treated with 15 medicinal plants (40 use reports) and there is much more consensus on the use of some of these species, compared with diabetes. Two species especially may be distinguished:* Maytenus ilicifolia* (15 UR) and* Aristolochia triangularis* (10 UR). High levels of cholesterol have a shorter trajectory of being treated with medicinal plants than blood cleansing but longer than diabetes. There are 10 species (28 UR) used to treat high levels of cholesterol, but two of them were most often reported:* Sorocea bonplandii* (9 UR) and* Maytenus ilicifolia* (7). This comparison of the consistency of/consensus on used plants is yet another point in favour of the idea that Paraguayan people are in a constant search for adequate medicinal plants to treat diabetes and, to a lesser extent, high levels of cholesterol.

Another cluster of illnesses and corresponding plants used with yerba mate extract I expected to be observed was composed of heart problems, nervous tension, and hypertension. However, the results confirm this assumption only partly, which may be due to the uneven number of species used in the treatment of hypertension (19 species), heart problems (9), and nervous tension (9). The greatest similarity was between heart problems and nervous tension at 44%. Three introduced species were used to treat these three health problems: lemon grass, mint, and rosemary.

Similarities can be observed between plants used for kidney and urinary infections: seven different species are used for both health conditions (58% of similarity), of which the most important ones are* Bauhinia microstachya*,* Equisetum giganteum,* and* Phyllanthus niruri*.

### 3.4. Forms of Administration

There is a certain pattern to the administration of plants with* mate* and* tereré* in respect to the illnesses that are treated.* Mate* is a vehicle to ingest hot remedies (for “cold” syndrome) and plants which treat women's health conditions (vaginal discharge, menstrual regulation, and pregnancy prophylaxis). The convergence of plants used in both cases is at 50%. In five cases these are “hot” remedies such as* Aristolochia triangularis*,* Lippia alba*,* Malva parviflora*,* Matricaria chamomilla,* and* Sida cordifolia* used in both categories. Interestingly all panacea plants are employed with the hot* mate* drink, except* Verbena *spp. On the other hand, refreshing plants (for “hot” syndrome) are prepared with* tereré*. Most species used for urinary infections and kidney problems are used with* tereré* too. However, the similarity in these two medicinal categories: refreshing plants and urinary/kidney problems is very low, at 13%, which means that in both cases quite different plants are prepared together with the* tereré*. Schmeda-Hirschmann and Bordas [23] also observed that Paraguayan folk medicine recognizes “hot” and “cold” remedies, whose uses dictate the manner of preparation and administration, namely, either in* mate* or in* tereré*.

According to Goyke [26], some plants are associated with either the hot or the cold version because of their flavour. Bitter and sharp flavours such as wormwood and* anís* are preferred in hot drinks while smooth flavours like mint, lemon grass, and saffron are preferred with the cold version [26]. Among 97 medicinal plants used by Paraguayan migrants with the yerba mate beverage, 23 are distinguished by their specific flavour (some of them contain essential oils) or taste (bitter, sour, or sweet). Fourteen of these plants are used with* mate*, five with* tereré,* and four with both. Bitter and sharp flavoured plants are used more often in mate, for example,* Artemisia absinthium*,* Baccharis trimera* and* B*.* gaudichaudiana*,* Dysphania ambrosioides*,* Ruta chalepensis, *or* Tagetes minuta*. However, bitter plants are also used with* tereré*:* Hypochaeris chillensis*,* Lactuca virosa,* and* Verbena* spp. Sour tasting plants are used only with* tereré,* for example,* Begonia cucullata*.

## 4. Conclusions

Yerba mate* (Ilex paraguariensis)* is not considered a medicinal plant by Paraguayan migrants living in Misiones, Argentina, by its own medicinal virtues. This plant, however, is culturally and medicinally a very important conveyor for medicinal plant intake. The Paraguayan community in Misiones uses 97 plant species in hot and cold versions of the yerba mate beverage. Most of the plants are native to the south cone of South America and come from cultivation.

There is a certain pattern of administration of plants with* mate* and* tereré* in respect to the illnesses that are treated.* Mate* is a vehicle to ingest hot remedies (for “cold” syndrome) and plants which treat women's reproductive health. Refreshing plants (for “hot” syndrome) are always prepared with* tereré*. Most species used for urinary infections and kidney problems are used with* tereré* too. Some plants are associated with either the hot or the cold version because of their flavour and taste. Bitter and sharp flavoured plants are more often used in* mate*. Sour tasting plants are used only with* tereré*.

The plants are used for a wide array of medicinal categories from which the digestive system and humoral concept prevail—traditionally treated with medicinal plants. On the other hand, relatively new chronic health conditions: diabetes, hypertension, and high levels of cholesterol are also treated with a large number of species. Therefore, this traditional form of administration of medicinal plants proves to be receptive for treating new health problems. Newly incorporated medicinal plants, such as* Moringa oleifera *or* Annona muricata,* are applied predominantly or exclusively with the* mate* beverage. The question which remains unanswered is whether medicinal plants with pharmacological properties similar to yerba mate have an additive or synergetic function in the beverage.

## Figures and Tables

**Table 1:** List of medicinal plants used with *mate* and *tereré* beverages by Paraguayan migrants living in Misiones, Argentina.

Botanical name	Local name	Plant part used	Medicinal use	Mode of administration	Status	Number of use reports	Relative importance (RI)	Index of Agreement on Species (IAS)
*Acanthospermum australe* (Loefl.) Kuntze, Asteraceae, CTES0049658	Tapekue	Leaves, aerial parts	Ulcers, digestion problems	In mate	Native	5	13	0,75

*Achyrocline flaccida* (Weinm.) DC. Asteraceae, CTES0032301; *Achyrocline tomentosa* Rusby, Asteraceae, CTES0049806	Marcela, jate'i ka'a	Flower	Digestion problem, stomach ache, hot remedy	In mate	Native	17	26,5	0,875

*Acrocomia aculeata *(Jacq.) Lodd. ex Mart., Arecaceae	Coco	Leaves	Hypertension, digestion problem	In tereré	Native	3	18,5	0,5

*Allophylus edulis *(A.St.-Hil., A. Juss. & Cambess.) Radlk., Sapindaceae, CTES0026306	Kokû	Leaves	Cold remedy, digestion problem, heartburn, hapatitis, blood cleansing, hypertension, hangover, prophylactic	In tereré	Native	34	64,5	0,74

*Aloe arborescens* Mill., Xanthorrhoeaceae	Aloe de hoja angosta	Leaves	Cancer, heart problem	In mate	Introduced	3	18,5	0,5

*Aloysia citriodora* Palau, Verbenaceae, CTES0026156	Cedrón	Leaves	Heart problem	In mate	Native	1	9,5	0

*Aloysia gratissima* (Gillies & Hook.) Tronc., Verbenaceae, CTES0049841	Poleo de palo	Leaves	Digestion problem	In mate	Native	1	9,5	0

*Aloysia polystachya *(Griseb.) Moldenke, Verbenaceae, CTES0032292	Burrito	Leaves	Digestion problem, stomach ache, hypertension, prophylactic	In mate	Native	12	32	0,72

*Aloysia pulchra *(Briq.) Moldenke, Verbenaceae, CTES0049842	Poleo	Leaves	Infecctions	In mate	Native	1	9,5	0

*Anethum graveolens *L., Apiaceae, BA92101	Eneldo	Seeds	Digestion problem, stomach ache	In mate	Introduced	2	13	0

*Annona muricata* L., Annonaceae	Graviola	Leaves	Cancer	In mate	Introduced	2	9,5	1

*Aristolochia triangularis* Cham., Aristolochiaceae, CTES0026276	Ysypo milhombre(s)	Liana	Blood cleansing, urinary infection, hypertension, cramps, abortive, contraceptive, restores menstruation, venereal disease, digestion problem, infections, internal cold, rheumatism, cure-all, prophylactic	In mate	Native	28	100	0,55

*Artemisia absinthium *L., Asteraceae, CTES0030264	Ajenjo	Leaves	Stomach ache, digestion problem, diabetes, cure-all	In mate	Introduced	10	30	0,67

*Baccharis gaudichaudiana* DC., Asteraceae, CTES0026286	Chirca melosa	Leaves	Blood cleansing, digestion problem, stomach ache	In mate	Native	3	22,5	0

*Baccharis trimera *(Less.) DC., Asteraceae, CTES0032297	Jaguarete ka'a, carqueja	Leaves	Digestion problem, stomach ache, diabetes, high level of cholesterol, blood cleansing, hangover	In mate	Native	19	50,5	0,72

*Bauhinia forficata *Link, Fabaceae, CTES0049618	Pata del buey	Leaves	Kidney infection	In mate and tereré	Native	2	9,5	1

*Bauhinia microstachya *(Raddi) J.F.Macbr., Fabaceae, CTES0032148	Ka'i escalera, pata del buey'i	Liana	Urinary infection, liquid retention, diabetes	In mate and tereré	Native	7	22,5	0,67

*Begonia cucullata* Willd., Begoniaceae, BA92089	Agrial	Leaves	Aphtha	In tereré	Native	1	9,5	0

*Bidens pilosa *L., CTES0049811, *Bidens subalternans *DC., Asteraceae, CTES0026269	Amor seco, picón	Aerial parts	Prostate, diabetes, hot remedy	In mate	Native	3	28	0

*Boerhavia diffusa* L., Nyctaginaceae, CTES0055777	Ka'arurupe	Aerial parts	Stomach ache	In tereré	Introduced	1	9,5	0

*Campomanesia* sp., Myrtaceae, CTES0049691	Yva hái mi	Tree bark	Diabetes	In mate	Native	1	9,5	0

*Campomanesia xanthocarpa *(Mart.) O.Berg, Myrtaceae, CTES0055662	Guavira	Leaves	Diarrhea	In mate	Native	1	9,5	0

*Cecropia pachystachya *Trécul, Urticaceae, CTES0032286	Amba'y	Leaves, young shoots	Cough, bronchitis, rheumatism, gastritis	In mate	Native	5	31,5	0,25

*Cissus verticillata* (L.) Nicolson & C.E.Jarvis, Vitaceae, CTES0055762	Insulina	Leaves	Diabetes	In mate	Native	2	9,5	1

*Citrus* × *aurantium* L., Rutaceae, CTES0032134	Apepu	Leaves, fruit	Nervous tension, high level of cholesterol	In mate and tereré	Naturalized	2	18,5	0

*Citrus reticulata *Blanco, Rutaceae, CTES0032133	Mandarina	Leaves	Cold sore	In mate	Introduced	1	9,5	0

*Citrus sinensis* (L.) Osbeck, Rutaceae, CTES0032133	Naranja	Leaves	Nervous tension, headache, hypertension	In mate	Introduced	5	27,5	0,5

*Croton* sp., Euphorbiaceae, CTES0049687	Sangre de grado	Tree bark	Diabetes, lack of appetite	In mate	Native	2	18,5	0

*Cyclospermum leptophyllum* (Pers.) Sprague, Apiaceae, CTES0049517	Apio'i	Aerial parts	Cold remedy, hangover, cold sore	In tereré	Native	5	22,5	0,5

*Cymbopogon citratus *(DC.) Stapf, Poaceae, CTES0048733	Cedrón	Leaves	Heart problems, nervous tension, hypertension, digestion problems, prophylactic	In mate and tereré	Introduced	20	41	0,79

*Cynodon dactylon *(L.) Pers., Poaceae, CTES0025340	Gramilla	Root	Liquid retension	In tereré	Native	1	9,5	0

*Dolichandra unguis-cati *(L.) L.G. Lohmann, Bignoniaceae, CTES0049524	Uña de gato	Rhizome	Kidney infection, urinary problems, cold remedy	In tereré	Native	5	18,5	0,5

*Dysphania ambrosioides *(L.) Mosyakin & Clemants, Amaranthaceae, CTES0032130	Ka'arê	Leaves	Hot remedy	In mate	Introduced	3	9,5	1

*Elionurus muticus *(Spreng.) Kuntze, Poaceae, CTES0026294	Kapi'i cedrón	Leaves	Nervous tension	In mate	Native	1	9,5	0

*Equisetum giganteum *L., Equisetaceae, CTES0049521	Cola de caballo	Leaves	Kidney infection, urinary problems, liquid retention, kidney stones	In mate and tereré	Native	12	20,5	0,72

*Eriobotrya japonica *(Thunb.) Lindl., Rosaceae, CTES0032147	Níspero	Leaves, young shoots	Hypertension, all-cure	In mate and tereré	Introduced	2	18,5	0

*Eugenia pyriformis* Cambess. var. pyriformis, Myrtaceae, CTES0048743	Yva hái	Leaves	Diabetes, hypertension, high level of cholesterol	In mate and tereré	Native	7	18,5	0,66

*Eugenia uniflora *L., Myrtaceae, CTES0026288	Ñangapiry, pitanga	Leaves	Hypertension, cold remedy	In mate and tereré	Native	11	18,5	0,82

*Euphorbia prostrata* Aiton, Euphorbiaceae	Tupasy kamby	Aerial parts	Liquid retension, blood cleansing	In mate	Native	2	18,5	0

*Euphorbia serpens *Kunth, Euphorbiaceae, CTES0026291	Tupasy kamby	Aerial parts	Cold remedy, vaginal discharge	In mate	Native	2	18,5	0

*Foeniculum vulgare *Mill., Apiaceae, CTES0049511	Aipo, hinojo	Aerial parts	Muscle pains, overheated stomach	In mate and tereré	Introduced	2	18,5	0

*Gleditsia amorphoides* (Griseb.) Taub., Fabaceae, CTES0026088	Ñuatî kurusu	Tree bark	Hypertension, infection	In mate	Native	2	18,5	0

*Gomphrena celosioides *Mart. f.* roseiflora *(Chodat & Hassl.) Pedersen, Amaranthaceae, CTES0055763	Perudilla	Aerial parts	Cold remedy, cold sore	In tereré	Native	4	13	0,67

*Guarea macrophylla* Vahl subsp.* spiciflora* (A. Juss.) T.D. Penn., Meliaceae, CTES0026272	Cedrillo	Trunk	Inernal jab and wound	In mate and tereré	Native	3	9,5	1

*Gynerium *spp., Poaceae	Caña brava	Root	Blood cleansing	In mate	Native	1	9,5	0

*Hemionitis tomentosa *(Lam.) Raddi, Pteridaceae, CTES0049653	Doradilla	Leaves	Vaginal discharge, irregular menstruation, bladder infection, blood cleansing	In mate	Native	4	31,5	0

*Heteropterys glabra *Hook & Arn., Malpighiaceae, CTES0026280	Tilo	Leaves, flower	Heart problem, nervous tension	In mate	Native	2	18,5	0

*Hypochaeris chillensis* (Kunth) Hieron., Asteraceae, CTES0026099	Achicoria silvestre	Root, leaves	Cold remedy	In tereré	Native	6	9,5	1

*Jacaranda micrantha* Cham., Bignoniaceae, CTES0026265	Karoba	Tree bark	Blood cleansing, cure-all	In mate	Native	7	18,5	0,83

*Jacaratia spinosa *(Aubl.) A.DC., Caricaceae, CTES0032144	Jakarati'a	Leaves	Hypertension	In mate	Native	1	9,5	0

*Lactuca virosa* Habl., Asteraceae, CTES0030265	Lechuga japonesa	Leaves	Hypertension	In tereré	Introduced	1	9,5	0

*Lippia alba* (Mill.) N.E.Br. ex Britton & P.Wilson, Verbenaceae, BA92110	Salvia	Leaves, flower	Menstrual pain, stomach pain, hot remedy, prophylactic, all-cure	In mate	Native	8	50,5	0,43

*Lippia brasiliensis* (Link) T.R.S. Silva, Verbenaceae, CTES0049516	Jate'i ka'a ka'aguy	Leaves	Abdomen pain, hot remedy, prophylactic	In mate	Native	3	28	0

*Malva parviflora* L., Malvaceae, CTES0049514	Malva de castilla	Leaves	Prophylactic during pregnancy, hot remedy	In mate	Introduced	2	18,5	0

*Mangifera indica* L., Anacardiaceae	Mango	Leaves, seed	Hypertension, constipation	In mate and tereré	Introduced	4	18,5	0,67

*Matricaria chamomilla* L., Asteraceae, CTES0026285	Manzanilla	Inflorescence	Menstrual pain, stomach pain, hot remedy, infections, prophylactic	In mate	Introduced	9	50,5	0,5

*Maytenus ilicifolia* Mart. ex Reissek, Celastraceae, CTES0048746	Kangorosa	Leaves, root, bark	Blood cleansing, high level of cholesterol, hypertension, internal infections, ulcers, abdomen pain, urinary infection, hot remedy	In mate	Native	30	77	0,76

*Melilotus *sp., Fabaceae	Alfalfa	Aerial parts	Rheumatism, eye problem	In mate and tereré	Introduced	2	18,5	0

*Melissa officinalis* L., Lamiaceae, CTES0032129	Toronjil	Leaves	Heart problem, cold remedy	In mate and tereré	Introduced	2	18,15	0

*Mentha *sp., Lamiaceae [*Mentha spicata* L. CTES0030909]	Menta, menta'i	Leaves	Nervous tension, heart problem, hypertension, headache, intestinal problems, abdomen pain, hot remedy, cold remedy, yerba mate additive	In mate and tereré	Introduced	16	58,25	0,53

*Moringa oleifera* Lam., Moringaceae, CTES0049652	Moringa	Leaves	Diabetes, high level of cholesterol, cancer, ulcers, digestion problem, kidney infection, urinary problem, prophylactic, all-cure	In mate and tereré	Introduced	15	69,25	0,43

*Morus alba* L., Moraceae, CTES0049525	Mora	Leaves	Hypertension, excessive libido	In mate and tereré	Introduced	2	18,5	0

*Myrocarpus frondosus* Allemao, Fabaceae	Incienso	Tree bark	Ulcers	In mate	Native	1	9,5	0

*Ocimum* cf.* basilicum* var.* anisatum* Benth, Lamiaceae, CTES0030915	Anís	Leaves	Prophylactic	In mate	Introduced	3	18,5	1

*Panicum tricholaenoides* Steud., Poaceae, CTES0032493	Cola de caballo	Leaves	Kidney infection	In mate and tereré	Native	2	9,5	1

*Parietaria debilis* G. Forst., Urticaceae, CTES0055778	Ka'a piky	Aerial parts	Cold remedy, cold sore, nervous tension,	In tereré	Native	14	31,5	0,85

*Parietaria judaica* L., Urticaceae, CTES0049674	Buscapina	Leaves	Digestion problem	In mate	Introduced	1	9,5	0

*Passiflora alata* Curtis, Passifloraceae, CTES0032140	Mburukuja	Leaves	Nervous tension	In tereré	Native	1	9,5	0

*Peperomia circinnata *Link, Piperaceae, CTES0049675	Jatevu ka'a	Entire plant	Hypertension	In tereré	Native	2	9,5	1

*Persea americana* Mill., Lauraceae	Palta	Seeds	Kidney infection	In mate	Naturalized	1	9,5	0

*Persea americana* var. *drimyfolia *(Cham. & Schltdl.) S.F.Blake, Lauraceae, BA92058	Palta anís	Leaves	Heart problem, liver problem, cold remedy	In mate and tereré	Naturalized	3	28	0

*Petroselinum crispum* (Mill.) Fuss, Apiaceae, CTES0049501	Perejíl	Leaves	Cold remedy	In tereré	Introduced	1	9,5	0

*Phyllanthus niruri* L., Phyllanthaceae, CTES0026299	Rompepiedras, quebrapiedra, para'parai	Aerial parts	Kidney infection, urinary problems, liquid retention	In mate and tereré	Native	7	17	0,67

*Piper mikanianum* (Kunth) Steud., Piperaceae, CTES0032289	Pariparoba	Leaves	Heart problem, abdomen pain, hot remedy	In mate	Native	5	28	0,5

*Plantago australis* Lam., Plantaginaceae, BA92072	Llantén	Leaves	Flu, sore throat, infections, cold sore, blood cleansing, prophylactic, cure-all	In mate	Native	10	54,5	0,33

*Pluchea sagittalis* Less., Asteraceae, CTES0048748	Yerba del lucero	Leaves	Digestion problem	In mate	Native	1	9,5	0

*Rollinia salicifolia* Schltdl., Annonaceae, CTES0026257	Aratiku	Leaves, flower	Hypertension, diabetes	In mate	Native	4	18,5	0,67

Rosa sp., Rosaceae, CTES0049673	Rosa	Flower	Constipation	In mate	Introduced	1	9,5	0

*Rosmarinus officinalis* L., Lamiaceae, CTES0049845	Romero	Leaves	Heart problem, nervous tension, abdomen pain, stomach pain, high level of cholesterol, hypertension, blood cleansing, hot remedy, headache, imporves memory, prophylactic, all-cure, yerba mate additive	In mate	Introduced	32	84,5	0,65

*Ruta chalepensis* L., Rutaceae, CTES0026289	Ruda	Leaves	Sore throat, prophylactic	In mate	Introduced	3	18,5	0,5

*Scoparia dulcis* L., Plantaginaceae, CTES0049830	Typycha kuratû	Aerial parts	Digestion problem, stomach ache, diabetes	In mate	Native	3	22,5	0

*Sechium edule* (Jacq.) Sw., Cucurbitaceae, CTES0026263	Chuchu, xuxu	Leaves	Hypertension	In mate	Naturalized	1	9,5	0

*Sida cordifolia* L., Malvaceae, CTES0026266	Malva blanca	Leaves, flower	Vaginal discharge, vaginal infection, irregular menstruation, menstrual pain, prophylactic during pregnancy, hot remedy, blood cleansing, kidney infection, urinary problem	In mate	Native	19	62	0,55

*Smallanthus connatus* (Spreng.) H.Rob., Asteraceae, CTES0049444	Jaguarete po	Leaves	Diabetes, high level of cholesterol, stomach ache	In mate and tereré	Native	7	28	0,67

*Solanum sisymbriifolium* Lam., Solanaceae, CTES0032288	Ñuatî pytâ	Root	Urinary infection	In tereré	Introduced	1	9,5	0

*Solidago chilensis* Meyen, Asteraceae, CTES0049655	Teju ka'a, yerba del lagarto	Aerial parts	Prophylactic	In mate	Native	1	9,5	0

*Sorocea bonplandii* (Baill.) W.C. Burger, Lanj.&Wess. Boer, Moraceae, CTES0026282	Ñandypa	Root, leaves	High level of cholesterol, diabetes, obesity, blood cleansing	In mate	Native	15	32	0,78

*Stachytarpheta cayennensis *(Rich.) Vahl, Verbenaceae, CTES0055765	Tatu ruguái, verbena tatu ruguái	Aerial parts	Prostate, male aphrodisiac	In mate and tereré	Native	2	13	0

*Stevia rebaudiana* (Bertoni) Bertoni, Asteraceae, CTES0049664	Ka'a he'ê	Leaves	Heartburn, diabetes, prophylactic	In mate	Native	4	28	0,33

*Styrax leprosus* Hook. & Arn., Styracaceae, CTES0026167	Carne de vaca	Tree bark	Diabetes, high level of cholesterol	In mate	Native	4	18,5	0,67

*Tabernaemontana catharinensis* A.DC., Apocynaceae, CTES0049506	Horquetero	Leaves	Diabetes, high level of cholesterol	In mate	Native	2	18,5	0

*Tagetes minuta* L., Asteraceae, CTES0026292	Suico	Leaves, flower	Intestinal parasites, empacho (folk illness)	In mate	Native	2	18,5	0

*Tanacetum parthenium* (L.) Sch. Bip., Asteraceae, CTES0049661	Manzanilla guasu	Flower	Stomach ache	In mate	Introduced	1	9,5	0

*Urera baccifera* (L.) Gaudich. ex Wedd., Urticaceae, CTES0026278	Ortiga grande	Root	Urinary problem, liquid retension, blood cleansing	In tereré	Native	4	22,5	0,33

*Verbena litoralis* Kunth, Verbenaceae, CTES0026305; *Verbena montevidensis* Spreng., Verbenaceae, BA92064	Verbena	Leaves	Stomach ache, digestion problem, lack of appetite, fever, diabetes, cold remedy, blood cleansing, hangover, headache, cure-all	In tereré	Native	18	66	0,59

*Xanthium spinosum* L., Asteraceae, CTES0049666	Cepacaballo, abrojito	Leaves	Digestion problem, liver problem, cold remedy	In mate and tereré	Introduced	3	22,5	0

*Zea mays* L., Poaceae	Barba de choclo	Flower	Urinary problem	In mate	Introduced	2	9,5	1
